# Resilience level and its association with maladaptive coping behaviours in the COVID-19 pandemic: a global survey of the general populations

**DOI:** 10.1186/s12992-022-00903-8

**Published:** 2023-01-03

**Authors:** Martin C. S. Wong, Junjie Huang, Harry H. X. Wang, Jinqiu Yuan, Wanghong Xu, Zhi-Jie Zheng, Hao Xue, Lin Zhang, Johnny Y. Jiang, Jason L. W. Huang, Ping Chen, Zhihui Jia, Erlinda Palaganas, Pramon Viwattanakulvanid, Ratana Somrongthong, Andrés Caicedo, María de Jesús Medina-Arellano, Jill Murphy, Maria B. A. Paredes, Mellissa Withers, On behalf of the Association of Pacific Rim Universities

**Affiliations:** 1 JC School of Public Health and Primary Care, Faculty of Medicine, The Chinese University of Hong Kong, Hong Kong, China; 2 Centre for Health Education and Health Promotion, Faculty of Medicine, The Chinese University of Hong Kong, Hong Kong, China; 3 Department of Sports Science and Physical Education, The Chinese University of Hong Kong, Hong Kong, China; 4 School of Public Health, Sun Yat-Sen University, Guangzhou, China; 5 The Seventh Affiliated Hospital, Sun Yat-Sen University, Shenzhen, China; 6 Department of Epidemiology, School of Public Health, Fudan University, Shanghai, China; 7 Department of Global Health, School of Public Health, Peking University, Beijing, China; 8 Center for Experimental Economics in Education, Shaanxi Normal University, Xi’an, China; 9 School of Population Medicine and Public Health, Chinese Academy of Medical Sciences & Peking Union Medical College, Beijing, China; 10 North Ruijin Hospital, Shanghai Jiaotong University, Shanghai, China; 11 Institute of Management, University of the Philippines Baguio, Baguio, Philippines; 12 College of Public Health Sciences, Chulalongkorn University, Bangkok, Thailand; 13 Instituto de Investigaciones en Biomedicina iBioMed, Universidad San Francisco de Quito, Quito, Ecuador; 14 Colegio de Ciencias de La Salud, Escuela de Medicina, Universidad San Francisco de Quito, Quito, Ecuador; 15 Sistemas Médicos SIME, Universidad San Francisco de Quito, Quito, Ecuador; 16 Institute of Legal Research, National Autonomous University of Mexico (UNAM), Mexico, Mexico; 17 Department of Psychiatry, Faculty of Medicine, The University of British Columbia, Vancouver, Canada; 18 i3S-Instituto de Investigação E Inovação Em Saúde, Universidade Do Porto, Porto, Portugal; 19 Department of Population and Public Health Sciences, APRU Global Health Program, Keck School of Medicine, University of Southern California, Soto Street Building, MC 9239, Los Angeles, CA 90089-9239 USA

**Keywords:** COVID-19, Pandemic, Resilience, Maladaptive coping behaviours

## Abstract

**Background:**

The coronavirus disease 2019 (COVID-19) pandemic has induced a significant global concern on mental health. However few studies have measured the ability of individuals to “withstand setbacks, adapt positively, and bounce back from adversity” on a global scale. We aimed to examine the level of resilience, its determinants, and its association with maladaptive coping behaviours during the pandemic.

**Methods:**

The Association of Pacific Rim Universities (APRU) conducted a global survey involving 26 countries by online, self-administered questionnaire (October 2020-December 2021). It was piloted-tested and validated by an expert panel of epidemiologists and primary care professionals. We collected data on socio-demographics, socioeconomic status, clinical information, lifestyle habits, and resilience levels measured by the Brief Resilience Scale (BRS) among adults aged ≥ 18 years. We examined factors associated with low resilience level, and evaluated whether low resilience was correlated with engagement of maladaptive coping behaviours.

**Results:**

From 1,762 surveys, the prevalence of low resilience level (BRS score 1.00–2.99) was 36.4% (America/Europe) and 24.1% (Asia Pacific). Young age (18–29 years; adjusted odds ratio [aOR] = 0.31–0.58 in older age groups), female gender (aOR = 1.72, 95% C.I. = 1.34–2.20), poorer financial situation in the past 6 months (aOR = 2.32, 95% C.I. = 1.62–3.34), the presence of one (aOR = 1.56, 95% C.I. = 1.19–2.04) and more than two (aOR = 2.32, 95% C.I. = 1.59–3.39) medical conditions were associated with low resilience level. Individuals with low resilience were significantly more likely to consume substantially more alcohol than usual (aOR = 3.84, 95% C.I. = 1.62–9.08), take considerably more drugs (aOR = 12.1, 95% C.I. = 2.72–54.3), buy supplements believed to be good for treating COVID-19 (aOR = 3.34, 95% C.I. = 1.56–7.16), exercise less than before the pandemic (aOR = 1.76, 95% C.I. = 1.09–2.85), consume more unhealthy food than before the pandemic (aOR = 2.84, 95% C.I. = 1.72–4.67), self-isolate to stay away from others to avoid infection (aOR = 1.83, 95% C.I. = 1.09–3.08), have an excessive urge to disinfect hands for avoidance of disease (aOR = 3.08, 95% C.I. = 1.90–4.99) and transmission (aOR = 2.54, 95% C.I. = 1.57–4.10).

**Conclusions:**

We found an association between low resilience and maladaptive coping behaviours in the COVID-19 pandemic. The risk factors identified for low resilience in this study were also conditions known to be related to globalization-related economic and social inequalities. Our findings could inform design of population-based, resilience-enhancing intervention programmes.

## Introduction

The coronavirus disease 2019 (COVID-19) has exerted a substantial global impact in terms of its induced morbidity, mortality, and disruption on healthcare services. As of early March, 2022, there were a total of over 440 million confirmed cases and more than 5.9 million deaths in different countries over the globe [1]. Of those infected, at least 80% developed mild to moderate disease, and among those with severe disease, around 5% suffered from critical illness [2]. Recovered patients with persistent or new symptoms lasting for weeks or months could have long COVID [3], which consists of neuropsychiatric complications such as depression, insomnia, neurocognitive difficulties, and anosmia. The situation is still ongoing and evolving with emerging variants. It has been reported that people who were socio-economically vulnerable or groups in marginalized circumstances were more likely to experience negative health outcomes due to the COVID-19 pandemic [4, 5].

Recent evidence shows that the COVID-19 has exerted an unprecedented impact on globalization and health in terms of mobility, economy, and healthcare systems [6]. Although low- and middle-income countries (LMICs) tend to face greater challenges in controlling and mitigating COVID-19, high-income countries (HMICs) could also be struggling with the unmet healthcare demands due to higher population density and increased pace of globalization that may have precipitated the amplification of pandemic despite having sophisticated healthcare infrastructure and greater emergency preparedness [7]. It has been known that factors such as economic migration, increasing social inequalities, and changing modes of social capital with diminution of social cohesion that are associated with globalization may lead to mental health consequences [8]. In this respect, the resilience level of the general population is crucial to counteract its adverse consequences, including social isolation, economic policy uncertainty, a wide spectrum of mental disorders, and suicidal ideation [9]. Resilience refers to the ability of individuals to “withstand setbacks, adapt positively, and bounce back from adversity” [10]. From recent meta-analyses, the prevalence of anxiety, depression, and stress due to the pandemic has been estimated at 30% in the general population [11, 12]. On a positive note, psychological resilience has been consistently demonstrated as a protective factor against psychological distress especially during times of disasters [13], including the 2010 Haiti Earthquake [14] and the 2005 Hurricane Katrina in the United States (US) [15], and the 2013 Super Typhoon Haiyan in the Philippines [16]. It has been widely recognized that the application of the resilience framework could contribute to the formulation and development of preventive and health-promoting interventions [10]. It could buffer and reduce risk factors that cause negative consequences [17]. A substantial body of evidence showed that people are more prone to suffer from adverse psychological and mental health consequences during stressful events when they have inadequate resilience levels and coping abilities [18, 19].

Nevertheless, there is a scarcity of studies that examined the level of resilience of the general population during the COVID-19 pandemic. Previous evaluations are mainly performed in a certain country with a relatively modest sample size [20, 21]. In addition, it remains unknown whether individuals with low levels of resilience may engage in harmful coping behaviours during the pandemic – which could lead to a vicious cycle under the current environment of adversity. Although a comprehensive, multi-level model of cognitive processing, decision making, and behaviour has linked mental resilience and stress coping, whether this phenomenon can be observed in real-life setting is yet to be examined.

This study aimed to evaluate the global prevalence of low resilience in the general populations of 26 countries across 4 regions (Asia Pacific, America, Europe and Middle East). Also, we studied the factors independently associated with low resilience level, and tested the hypothesis that resilience is inversely associated with practice of maladaptive coping behaviours.

## Methods

### Study setting

The Association of Pacific Rim Universities (APRU) performed a global survey including 26 study sites by online, self-administered questionnaire. The Association is comprised of 60 leading universities from 19 economies of the Pacific Rim recognized worldwide for their research and academic excellence [22]. The APRU Global Health Programme is one of the most important initiatives of APRU, and was launched in 2007–08. It is designed to address regional and global health issues by expanding existing collaborative research efforts among Pacific Rim universities. This programme covers a wide range of academic disciplines, including non-communicable diseases such as mental health. The study sites include: 1). Asia Pacific countries or regions: Australia, mainland China, Hong Kong, India, Indonesia, Japan, Malaysia, New Zealand, Philippines, Russia, South Korea, Taiwan, and Thailand; 2). The Americas: Canada, Central America, Colombia, Ecuador, Mexico, and Peru; 3). European countries: the United Kingdom (UK), France, Germany, and Italy; and 4). The Middle East: Iraq; Saudi Arabia, and Oman.

### Participant recruitment

We established a team consisting of more than 20 investigators in different world regions who disseminated surveys to the general population of their own country via various channels, including a website linkage sent through emails and different social media platforms. We recruited study participants by circulation of the survey link from October 2020 to December 2021. The investigators utilized their networks to invite all eligible participants through snowball sampling, targeting the general populations. All subjects aged 18 years or above who are capable to comprehend the study and provide informed consent were eligible to participate. The online platform used is a secured software which ensures confidentiality of information collected. The database thus produced was password encrypted, and only research personnel may access the data. The data were stored in a secure place not accessible to other people. The study was approved by the Survey and Behavioural Research Ethics Committee of the Chinese University of Hong Kong (SBRE-20–035), which covered ethics clearance in all the study sites.

### Survey instruments

The survey was pilot-tested and validated by an expert panel of epidemiologists, and primary care professionals and physicians. It was available in eight different languages. We collected data on the sociodemographic variables, socioeconomic status, clinical information, lifestyle habits, and resilience levels measured by the Brief Resilience Scale (BRS) [23]. It consists of six questions and the participants were requested to choose one response for each question among five Likert scales, including “strongly disagree”, “disagree”, “neutral”, “agree” and “strongly agree”. Each question carries a maximum of 5 marks and the possible total score, by simple summation, ranges from 6–30. The BRS score was then derived by dividing the total score by 6, with a score of 1.00–2.99; 3.00–4.30 and 4.31–5.00 considered as low, normal, and high resilience, respectively. The original BRS was devised to measure an individual’s ability to “(1). bounce back or recover from stress; (2). to adapt to stressful circumstances; (3). to not become ill despite significant adversity; and (4). function above the norm despite stress or adversity” [24, 25]. It has a good internal consistency and test–retest reliability with Cronbach’s alpha of 0.80–0.91 [23]. Assume the proportion (p) of the primary outcome on resilience as 50% which gives the maximum sample size, at least 1,537surveys are needed to achieve a precision of 0.025 based on the formula: *N* = 1.96^2^ *p (1-p)/[precision squared].

### Statistical analysis

All data were entered and analysed by the IBM Statistical Package for Social Sciences version 25 (IBM SPSS 25). We performed a descriptive analysis of the study participants according to their demographic details, socioeconomic status and past medical history. The prevalence of low, normal, and high resilience levels were computed for participants Americas and Europe, Asia Pacific countries, and Middle East. We examined the factors associated with low resilience level (vs. normal and high resilience as a comparison group), including age, sex, years of education, location of residence (urban vs. rural), changes in private financial situation and body weight in the past 6 months, as well as the number of chronic conditions. Low resilience level was the outcome variable. Potential associated factors with *p* < 0.20 in bivariate analysis were included in a binary logistic regression model.

We also evaluated whether low resilience (BRS score of 1.00–2.99) was associated with unhealthy behaviours and risk factors, including substantial increase in alcohol consumption; use of more drugs than usual; purchasing drugs, herbs, supplements, or other treatments believed to be good for treating COVID-19; exercising less than before the pandemic; eating more unhealthy food than before the pandemic; performing self-isolation to avoid people that might infect the participants; having an excessive urge to wash and/or disinfect their hands to avoid becoming ill or transmission of diseases to others. We set up nine separate binary regression models with each unhealthy behaviour and risk factor as an outcome variable consecutively. The predictor tested for association with these unhealthy behaviours was low resilience level, whilst controlling for potential associated factors listed above. Given that the changes in the pandemic over time might potentially impact resilience, we also performed sensitivity analysis to examine whether risk factors identified in the main analysis remained significant after adjusting for the time frame variable. All two-sided *P* values < 0.05 were considered as statistically significant.

## Results

### Participant characteristics

**Fig. 1:**
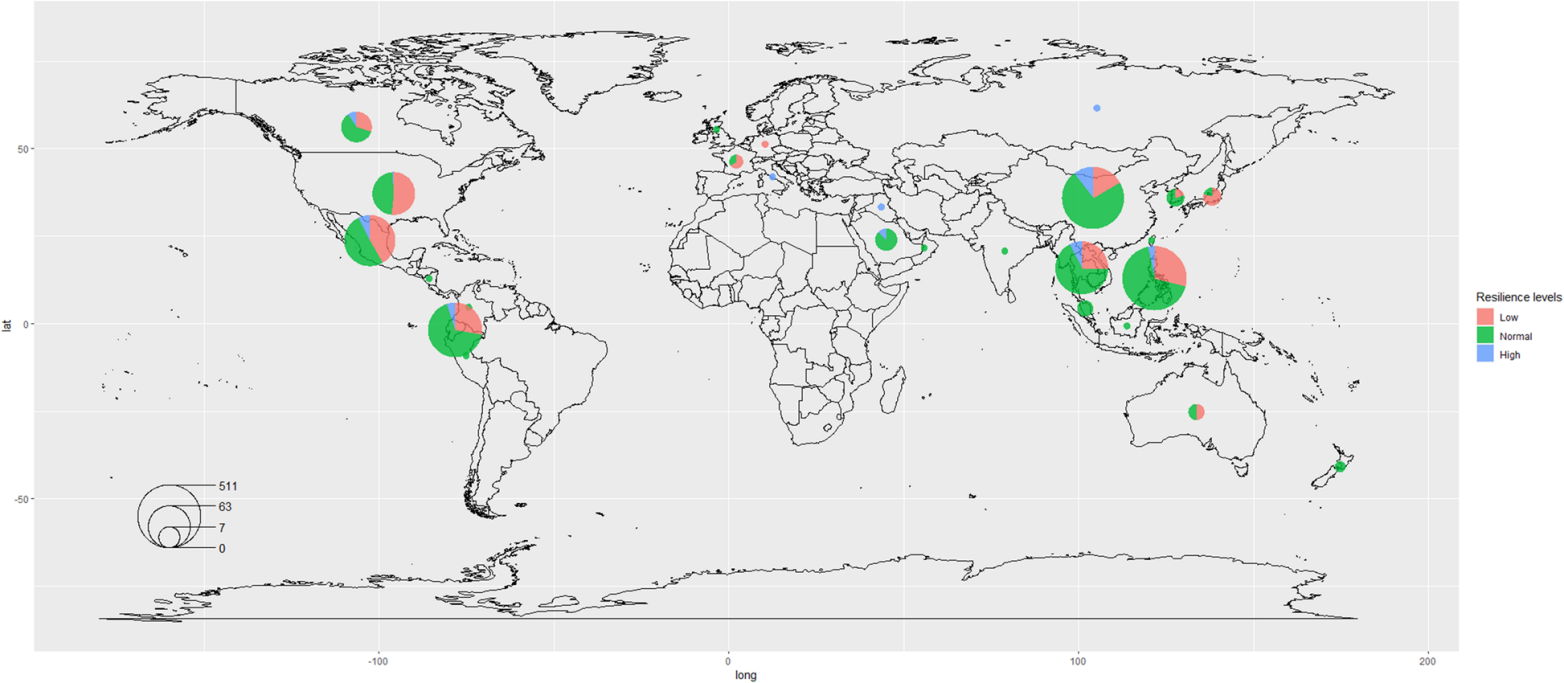
Geographical distribution of the study participants

We collected a total of 1,762 surveys and their geographical distribution is shown in Fig. 1. The most participants were aged 18–29 years (66.7%), female (66.2%), education > 12 years (80.9%), lived in urban areas (63.8%) and lived with their family members (80.3%). Close to half of the participants (41%) experienced weight gain and 23.7% reported having poorer financial situation in the past 6 months. The most common medical conditions reported were COVID-19 (16%), obesity (11.2%), mental health problems (8.6%), and respiratory diseases (7.9%). The proportion of having one and ≥ 2 medical conditions was 22.2% and 8.7%, respectively (Table 1).

**Table 1:** Participant characteristics

	**n (%)**
Age (years)	
18–29	1152 (66.7)
30–39	266 (15.4)
40–49	165 (9.5)
≥ 50	145 (8.4)
Sex	
Male	582 (33.8)
Female	1140 (66.2)
Years of Education	
0–9	73 (4.2)
10–12	262 (14.9)
> 12	1420 (80.9)
Residence	
Urban	1121 (63.8)
Rural	454 (25.8)
Others	182 (10.4)
Living status	
Alone	169 (9.6)
Family members	1411 (80.3)
Other people or none of the above	177 (10.1)
Private financial situation in the past six months	
Increased	305 (17.3)
Decreased	417 (23.7)
Remains the same	926 (52.6)
Do not know	110 (6.2)
Weight change over the past six months	
Increased	713 (40.9)
Decreased	242 (13.9)
Remained the same	710 (40.8)
Do not know	77 (4.4)
Past medical history	
Cardiovascular disease	45 (2.6)
Diabetes	43 (2.5)
Immunodeficiency^a^	54 (3.2)
Respiratory diseases	136 (7.9)
Liver diseases	30 (1.8)
Kidney diseases	31 (1.8)
Cancer in the past five years	29 (1.7)
Sickle cell diseases	27 (1.6)
Obesity (Body mass index ≥ 30 kg/m^2^)	192 (11.2)
Mental health issues^b^	151 (8.6)
Tested positive for COVID-19	171 (16)
Past medical history^c^	
0	1216 (69)
1	392 (22.2)
≥ 2	154 (8.7)

Missing responses were excluded in individual characteristics

^a^Including those who took medication that suppresses the immune system

^b^Mental health issues include mania/bipolar disorders, psychotic disorders (including schizophrenia), post-traumatic stress disorder, eating disorder, obsessive compulsive disorder, substance abuse and addiction disorder, attention disorder (Attention Deficit Disorder [ADD] or Attention Deficit Hyperactivity Disorder [ADHD]), somatoform disorder, personality disorder, autism spectrum disorder, cognitive disorder/dementia

^c^Past medical history includes: cardiovascular disease, diabetes, immunodeficiency, respiratory diseases, liver diseases, kidney diseases, cancer in the past five years, sickle cell diseases, obesity (BMI ≥ 30 kg/m^2^), mental health issues; and COVID-19

### Resilience levels and their associated factors

Among the study participants, around 27.2%, 66.4% and 6.4% were assessed as having low, normal, and high resilience, respectively (Table 2). The Americas and Europe had a higher proportion of individuals having low resilience (36.4%) than those in Asia Pacific countries (24.1%). Young age (18–29 years; range of adjusted odds ratio [aOR] = 0.31–0.58 in older age groups), female gender (aOR = 1.72, 95% C.I. = 1.34–2.20), poorer financial satiation in the past 6 months (aOR = 2.32, 95% C.I. = 1.62–3.34), the presence of one (aOR = 1.56, 95% C.I. = 1.19–2.04) and two or more (aOR = 2.32, 95% C.I. = 1.59–3.39) medical conditions were associated with low resilience level (Table 3).

**Table 2:** Resilience levels across different countries/regions

	**Resilience level**		
	**Low (1.00–2.99)**	**Normal (3.00–4.30)**	**High (4.31–5.00)**
All participants	479 (27.2%)	1,170 (66.4%)	113 (6.4%)
Americas and Europe	171 (36.4%)	272 (57.9%)	27 (5.7%)
Asia Pacific	304 (24.1%)	875 (69.3%)	84 (6.7%)
Middle East	4 (13.8%)	23 (79.3%)	2 (6.9%)

**Table 3:** Factors associated with low resilience level (BRS score 1.00–2.99)

	**n (%)**	**cOR (95% C.I.)**	***p***	**aOR (95% C.I.)**	***p***
Age			< 0.001		< 0.001
18–29	380 (33.0%)	1.0 (referent)		1.0 (referent)	
30–39	52 (19.5%)	0.49 (0.36—0.69)	< 0.001	0.58 (0.41—0.81)	0.002
40–49	21 (12.7%)	0.30 (0.18—0.48)	< 0.001	0.31 (0.19—0.51)	< 0.001
≥ 50	23 (15.9%)	0.38 (0.24—0.61)	< 0.001	0.43 (0.26—0.69)	0.001
Sex					
Male	121 (20.8%)	1.0 (referent)		1.0 (referent)	
Female	348 (30.5%)	1.67 (1.32—2.12)	< 0.001	1.72 (1.34—2.20)	< 0.001
Years of Education			0.001		0.100
0–9	21 (28.8%)	1.18 (0.70—1.99)	0.530	1.35 (0.77—2.37)	0.290
10–12	96 (36.6%)	1.69 (1.28—2.23)	< 0.001	1.37 (1.00—1.87)	0.050
> 12	362 (25.5%)	1.0 (referent)		1.0 (referent)	
Residence			0.150		0.870
Urban	289 (25.8%)	1.0 (referent)		1.0 (referent)	
Rural	139 (30.6%)	1.27 (1.00—1.62)	0.050	1.03 (0.79—1.33)	0.850
Others	51 (28.0%)	1.12 (0.79—1.59)	0.520	1.10 (0.75—1.62)	0.610
Private financial situation in the past six months			< 0.001		< 0.001
Increased	59 (19.3%)	1.0 (referent)		1.0 (referent)	
Decreased	161 (38.6%)	2.62 (1.86—3.71)	< 0.001	2.32 (1.62—3.34)	< 0.001
Remains the same	220 (23.8%)	1.30 (0.94—1.79)	0.110	1.33 (0.95—1.86)	0.097
Don’t know	38 (34.5%)	2.20 (1.36—3.57)	0.001	1.46 (0.86—2.46)	0.160
Weight change over the past six months			< 0.001		0.026
Increased	206 (28.9%)	1.0 (referent)		1.0 (referent)	
Decreased	86 (35.5%)	1.36 (1.00—1.85)	0.053	1.38 (0.99—1.91)	0.056
Remains the same	157 (22.1%)	0.70 (0.55—0.89)	0.003	0.83 (0.64—1.07)	0.160
Don’t know	28 (36.4%)	1.41 (0.86—2.30)	0.170	1.22 (0.73—2.05)	0.450
Number of chronic conditions^a^			< 0.001		< 0.001
0	296 (24.3%)	1.0 (referent)		1.0 (referent)	
1	122 (31.1%)	1.40 (1.09—1.81)	0.008	1.56 (1.19—2.04)	0.001
≥ 2	61 (39.6%)	2.04 (1.44—2.89)	< 0.001	2.32 (1.59—3.39)	< 0.001

*cOR* Crude odds ratio, *aOR* Adjusted odds ratio, *C.I.* Confidence interval, *BRS* Brief Resilience Score; those variables in univariate analysis with *p* < 0.20 to be included as predictors in multivariate analysis

^a^Chronic conditions include cardiovascular disease, diabetes, immunodeficiency, respiratory diseases, liver diseases, kidney diseases, cancer in the past five years, sickle cell diseases, obesity (BMI ≥ 30 kg/m^2^), mental health issues; and COVID-19

### Association between low resilience level and maladaptive coping behaviours

Individuals with low resilience were significantly more likely to consume substantially more alcohol than usual compared to the normal or high resilience individuals (aOR = 3.84, 95% C.I. = 1.62–9.08), take considerably more drugs than usual (aOR = 12.1, 95% C.I. = 2.72–54.3), bought drugs, herbs, supplements or other treatments believed to be good for treating COVID-19 (aOR = 3.34, 95% C.I. = 1.56–7.16), exercise less than before the pandemic (aOR = 1.76, 95% C.I. = 1.09–2.85), consume more unhealthy food than before the pandemic (aOR = 2.84, 95% C.I. = 1.72–4.67), self-isolate to stay away from others due to the fear of getting infected (aOR = 1.83, 95% C.I. = 1.09–3.08), experience an excessive urge to wash and/or disinfect hands again and again for avoidance of illness from germs or contamination (aOR = 3.08, 95% C.I. = 1.90–4.99) and transmission to other people (aOR = 2.54, 95% C.I. = 1.57–4.10) (Table 4). No variable interactions and multicollinearity were detected in both regression models. Sensitivity analysis by stratification of the time frame variable (i.e., date of questionnaire completion) and its inclusion in the multivariate regression analysis did not change the conclusions made.

**Table 4:** The association between resilience level and health behaviours

Resilience level	n (%)	cOR (95% C.I.)	*p*	aOR (95% C.I.)	*p*
Consumption of substantially more alcohol than usual					
1.00–2.99	107 (29.7%)	4.07 (1.90—8.72)	< 0.001	3.84 (1.62—9.08)	0.002
3.00–4.30	174 (20.5%)	2.49 (1.18—5.25)	0.017	2.68 (1.17—6.16)	0.020
4.31–5.00	8 (9.4%)	1.0 (referent)	< 0.001	1.0 (referent)	0.004
Consumed considerably more drugs (e.g., tranquilizers, hypnotics or stimulants) than usual					
1.00–2.99	83 (25.2%)	12.99 (3.12—54.05)	< 0.001	12.15 (2.72—54.29)	0.001
3.00–4.30	63 (8.0%)	3.34 (0.80—13.92)	0.098	3.27 (0.74—14.41)	0.120
4.31–5.00	2 (2.5%)	1.0 (referent)	< 0.001	1.0 (referent)	< 0.001
Bought drugs, herbs, supplements or other treatments heard being good for treating COVID-19					
1.00–2.99	112 (31.5%)	4.05 (1.96—8.35)	< 0.001	3.34 (1.56—7.16)	0.002
3.00–4.30	181 (20.8%)	2.31 (1.14—4.68)	0.021	1.94 (0.93—4.04)	0.077
4.31–5.00	9 (10.2%)	1.0 (referent)	< 0.001	1.0 (referent)	< 0.001
Exercised less than before the pandemic					
1.00–2.99	304 (69.7%)	2.55 (1.63—3.97)	< 0.001	1.76 (1.09—2.85)	0.022
3.00–4.30	585 (56.4%)	1.43 (0.95—2.16)	0.090	1.13 (0.73—1.76)	0.580
4.31–5.00	47 (47.5%)	1.0 (referent)	< 0.001	1.0 (referent)	0.003
Ate more unhealthy food than before the pandemic					
1.00–2.99	288 (65.0%)	4.24 (2.68—6.71)	< 0.001	2.84 (1.72—4.67)	< 0.001
3.00–4.30	503 (48.1%)	2.12 (1.37—3.26)	0.001	1.73 (1.09—2.75)	0.020
4.31–5.00	32 (30.5%)	1.0 (referent)	< 0.001	1.0 (referent)	< 0.001
Self-isolate to stay away from other people to avoid getting infected					
1.00–2.99	367 (82.7%)	2.00 (1.23—3.25)	0.005	1.83 (1.09—3.08)	0.023
3.00–4.30	763 (74.2%)	1.21 (0.78—1.88)	0.410	1.13 (0.71—1.80)	0.610
4.31–5.00	74 (70.5%)	1.0 (referent)	0.001	1.0 (referent)	0.005
Have had the excessive urge to wash and/or disinfect my hands again and again so that I do not become ill from germs or contamination					
1.00–2.99	331 (74.0%)	4.08 (2.61—6.38)	< 0.001	3.08 (1.90—4.99)	< 0.001
3.00–4.30	596 (57.6%)	1.94 (1.28—2.93)	0.002	1.70 (1.10—2.65)	0.018
4.31–5.00	42 (41.2%)	1.0 (referent)	< 0.001	1.0 (referent)	< 0.001
Have had the excessive urge to wash and/or disinfect my hands again and again so that I do not pass on germs or contamination to other people					
1.00–2.99	332 (76.0%)	3.63 (2.32—5.69)	< 0.001	2.54 (1.57—4.10)	< 0.001
3.00–4.30	615 (60.2%)	1.74 (1.15—2.62)	0.008	1.43 (0.93—2.21)	0.110
4.31–5.00	47 (46.5%)	1.0 (referent)	< 0.001	1.0 (referent)	< 0.001

Health behaviours: “not at all” and “seldom” as one group (referent group), with “neutral”, “often” and “very much” as one group (being tested for association); aOR: controlled for age group, gender, years of education, residence, financial situation, weight change over the past six months, and the “presence of 0, 1 or ≥ 2 medical conditions listed in Table 3” as covariates in the final regression equation

*cOR* Crude odds ratio, *aOR* Adjusted odds ratio, *C.I.* Confidence interval

## Discussion

From this global survey with 1,762 completed questionnaires involving 26 countries or regions, we found that the overall proportion of individuals having a low resilience level was more than one-quarter of the population, with a higher proportion in the Americas and European countries than in Asia Pacific regions. Younger individuals, female participants, those with a poorer financial situation in the past 6 months, and people with multimorbidity were associated with a low resilience level. We also found that low resilience was associated with the practice of various unhealthy behaviours and risk factors, which could represent maladaptive coping lifestyles.

### Measurement of resilience levels

There are few surveys that have measured the resilience levels of the general population performed on a global scale. Killgore et al. (2020) conducted an online questionnaire which collected data from 1,004 English speaking participants during the third week of the COVID-19 stay-at-home guidance in all 50 states of the US [20]. They used the Connor-Davidson Resilience Scale (CD-RISC) [26] and other validated surveys, including the Beck Depression Inventory-II (BDI-II), the Zung Self-Rated Anxiety Scale (SAS), and the Multidimensional Scale of Perceived Social Support (MSPSS), to measure psychiatric symptoms [20]. They found a significant lower psychological resilience level during the first weeks of the COVID-19 lockdown in the US when compared with published normative data for CD-RISC, implying a possible adverse influence of the pandemic conferring acute alterations in emotional outlook and perceived support. Also, they demonstrated that low resilience was associated with poorer mental health outcomes such as severe depression, anxiety, worry about the effects of COVID-19, greater difficulty coping with emotional challenges, and suicidal ideation. Another study among 898 young adults aged 18 to 30 years in the US from April to May 2020, which is one month after declaration of a state of emergency, found that up to 72% of the study participants, of which 81.3% were women, had low resilience [21]. High resilience level (score ≥ 30 in CD-RISC-10) was significantly associated with lower depression, anxiety and post-traumatic stress disorders. The findings of these studies, in conjunction with our results, highlight the low resilience levels of the general populations and the importance to enhance it given its potential psychological consequences.

### Resilience levels across different regions

We found that higher proportion of participants in Western countries reported lower resilience level than in Asia Pacific regions. In general, Asian individuals were less likely to report high levels of mental health symptoms than individuals from European and American regions [21]. Furthermore, Asian and Latinx immigrants, compared to participants born in the U.S., are less likely to endorse psychological distress [27, 28]. It has been speculated that other experiences such as ethnic identity, social networking, family cohesion, and even religiosity could act as a protective factor for mental health [29, 30].

### Factors associated with lower resilience

We found that young participants aged less than 30 years suffered from lower resilience than the elderly population. Previous evidence from both Western and Asian countries demonstrated that younger people, e.g., 18–24 years old (and not exceeding 40), had the greatest increase in rates of psychological distress during the pandemic [31–34]. Younger age (under 35 years old) has been shown to be a significant mediator for stress-anxiety mediation models [35], implying that younger adults may be more vulnerable to stress and anxiety during this pandemic. In general, younger individuals fared the worst when they experienced depression, stress, and anxiety symptoms. An Australian survey conducted in April 2020 also found that younger people aged < 45 years were the most vulnerable group to psychological distress [33]. Loneliness and financial distress have been linked to poorer depression and anxiety outcomes, respectively, in younger adults. On the contrary, older adults demonstrated higher resilience than other age-groups, which could be due to their greater ability to savour life experiences [36, 37].

Furthermore, female individuals tend to have lower resilience during the pandemic. This is compatible with a recent comprehensive review on sex differences in resilience [38]. Females demonstrate increased vulnerability in times of stress, which could be attributed to gender-, sex hormone-, and sex chromosome-life span interactions in producing resilience. This gender difference could also be due to internalization of trauma, generalization of fear cues, anhedonia, passive coping, and blunting of corticosteroid response to stress among women [39], although the influence of sex on risk and resilience to stress could be complex that varies according to the type, timing and duration of the stressor as well as development with its associated changes in brain structure and function. The findings are in line with recent literature from the perspective of social inequality that suggests additional gendered vulnerabilities and stresses placed on women during the lockdown and thereafter, ranging from decreased health care access, increased unemployment, domestic violence, and higher risks of exposure and infection via work in health care industries [40–42].

Last but not least, people with multimorbidity were more likely to have low resilience score. Despite the fact that the massive global effort and resources directed to COVID-19 have completely overshadowed the pandemics of noncommunicable diseases in the twenty-first century, multimorbidity remains a crucial element of both diminished resistance to coronavirus infection and diminished resilience as found in this research. Individuals with multimorbidity suffer from physical challenges as well as social-psychological feelings of stress, anxiety, depression, loneliness, low self-esteem, social isolation, and changes in social roles [43]. Our findings indicate that their resilience level was likely to be low, which could be explained by the need for continuous efforts in maintaining healthy levels of functioning following adversity, which is a dynamic process but not a personality trait [44]. Given the possible widening of inequalities in both income and welfare, further research are necessary to illustrate whether the impact of inequality on the resilience of societies within developing economies may differ from that in industrialized nations during the course of globalization.

We also observed the presence of associations between low level of resilience and maladaptive coping behaviours such as low level of exercise, greater uptake of unhealthy food, increased consumption of alcohol and medications. The practice of these maladaptive coping behaviours represents various unhealthy lifestyles, which could be conceptualised as ‘industrial diseases’ that are related to poor diet, alcohol, gambling, drug and tobacco related diseases as these are directly associated with the vectors, i.e., the unhealthy commodity industries [45]. These industries are known to target those with least education, employment and income, and are known to target them during the COVID-19 pandemic, with a wide range of national and international policies that continue to ensure intergenerational disadvantage within countries and between countries. Therefore, it is highly likely that the association between low resilience and maladaptive coping behaviours observed in our study is mediated by the activities of these unhealthy commodity industries. This further calls for a need to shape the policy strategies for mitigating the negative impact of these unhealthy commodity industries, and for minimising the resultant impact of highly ineffective and inequitable policies among people across the globe.

### Strengths and weaknesses of the study

This survey involved a large number of countries and represents a global collaboration from researchers in various study sites. The survey was devised and pilot-tested by an expert panel, and we used a published, validated resilience survey with good internal consistency and test–retest reliability. Nevertheless, there are several limitations that should be addressed. Firstly, we are unable to capture the response rate as this consecutive sampling strategy did not provide the number of participants who received the survey invitation. Furthermore, casual relationships between low resilience and the unhealthy behaviours could not be inferred, as there is a possibility of reverse causality in this cross-sectional study. In addition, the survey responses were received from a long period of time lasting for approximately two years. The number of new COVID-19 cases and mortality due to the severe acute respiratory syndrome coronavirus 2 (SARS-CoV-2) fluctuated with time, and it is unknown whether the severity of the pandemic, its related public health measures, and the changing social distancing measures in different COVID-19 waves might exert an impact on the resilience levels. Furthermore, the generalizability of our study findings to other settings should be interpreted with caution, as we did not adopt a random sampling strategy and not all countries provided a large number of responses. Selection bias might exist as the current survey tended to attract participants who were regular internet users. Also, this study did not evaluate the reasons of low resilience among participants with the associated factors, and the mechanistic aspects of how low resilience might lead to unhealthy behaviours examined in this survey. Further in-depth work is required to examine the detailed pathways where resilience might influence personal behaviours in the pandemic.

### Implications for health and practice

Our study findings have identified a subgroup of individuals who may be at a higher risk for low resilience level. In particular, it is worrying that resilience is not only a stand-alone observation – it could also be closely associated with more unhealthy behaviours as demonstrated by results of this global survey. Interventions targeting these at-risk participants are needed. For instance, a previous survey [20] identified that more time spent on outdoors in the sunshine for at least 10 min; daily exercise; more extensive support from family and friends; care from a close significant other; and improvement of spiritual health such as prayers could be predictive of better resilience. Physicians could offer anticipatory care during their clinical consultations to provide resources related to interventions for patients with low resilience, such as the adoption of a holistic approach and behavioural modification to enhance self-efficacy. Proactive measures to improve and sustain resilience, including building of coping skills and implementation of social support networks, could be crucial [46]. Policy-makers should consider community-based programmes that could target these at-risk individuals by evidence-based resilience-enhancing strategies through a concerted, multidisciplinary effort, as interventions to improve resilience is often multi-pronged.

From a global health perspective, the risk factors identified for low resilience in this study, such as advanced age, female gender, poor financial situation, and the presence of chronic conditions, and the consequences of low resilience levels that spans from food consumption to social engagement were known to be related to globalization-related economic and social inequalities [47]. The increasing gaps in social protection alongside the widening of inequalities across different socio-economic strata within and between countries have been revealed and exacerbated by the COVID-19 pandemic [48, 49]. Public health and social welfare systems may therefore need to be re-oriented with a joint focus to tackle health inequalities following the WHO’s Health in All Policies approach. This may warrant multi-sectorial efforts to ensure the timely and equitable delivery of appropriate, accessible, and affordable health and social care products to groups in socioeconomically vulnerable or marginalized circumstances who are more likely to suffer from negative mental health outcomes and poor lifestyle habits.

## Conclusions

We found that the Americas and Europe had a higher proportion of individuals having low resilience than those in Asia Pacific countries. Factors that were independently associated with low resilience level included young age, female gender, poorer financial satiation, and the presence of medical conditions. We suggested that resilience was inversely associated with practice of maladaptive coping behaviours in the COVID-19 pandemic. We recommend future studies to identify effective interventional programmes for vulnerable subjects with different characteristics, and the survey should be regularly repeated to capture trends of resilience levels over time.

## Data Availability

The datasets used and/or analysed during the current study are available from the corresponding author on reasonable request.

## Abbreviations

APRU: Association of Pacific Rim Universities
COVID-19: Coronavirus disease 2019
SARS-CoV-2: Severe acute respiratory syndrome coronavirus 2
CD-RISC: Connor-Davidson Resilience Scale
BRS: Brief Resilience Scale
aOR: Adjusted odds ratio
